# Supplementary material for: The impact of sampling bias on viral phylogeographic reconstruction
Source: PLOS Glob Public Health. 2022 Sep 28;2(9):e0000577. doi: 10.1371/journal.pgph.0000577 (PMC10021582; doi:10.1371/journal.pgph.0000577)
Supplement: S1 Text — (PDF) [file pgph.0000577.s001.pdf]

Supplementary Material

Supplementary Material for Liu et al. *"The impact of sampling bias on viral phylogeographic reconstruction"*

Absolute and Relative Reconstruction Accuracy

Here we present complementary results and sensitivity analyses in which we choose different values for fixed parameters and trees. S1 Fig shows the absolute and relative accuracy for the true trees displayed in Fig 4A and Fig 4C, in parallel with Fig 1B and Fig S1D. Whereas in the main text we downsampled many times from the same fixed true tree, in S2 Fig we show the absolute reconstruction accuracy and how it varies with sampling bias using 25 true trees for each choice of the migration rate; we downsample 50 times for each tree.

Each edge in the true tree has two endpoints, that is, the parent node of the edge near the root and the child node near the tips. For an edge in a downsampled tree of a true tree, each node has a true location from simulation and a reconstructed location (a likelihood of being location A) from the function `ace`. The state of the edge with the true locations at the endpoints can be A-A, A-B, B-A or B-B. We count an edge with a location-A parent node and a location-A child node as one edge in the state A-A and analogously for the other states. Whereas the state of the edge with reconstructed locations at the endpoints is computed by multiplying the likelihood of the parent node being at location A (or B) and the the likelihood of the child node being at location A (or B). For example, if the parent node has a likelihood 0.3 of being at location A and the child node has a likelihood 0.1 of being at location A, then we count the edge as 0.03 edge in the state A-A, 0.27 edge in the state A-B, 0.07 edge in the state B-A and 0.63 edge in the state B-B. We compute the number of edges with true locations at endpoints in each state in downsampled trees with different proportions of location-A tips for the true trees simulated with different migration rates. We also compute the difference of subtracting the number of edges with true locations at endpoints from the number of edges with reconstructed locations at endpoints in each state for the true trees. The results are displayed in S3 Fig and S4 Fig.

Furthermore, in the main text, we used a neutral branching process in which the branching and death rates did not depend on the location. Here, we simulate trees with non-neutral speciation rates. S5 Fig displays the absolute and relative accuracy for two true trees simulated with $\lambda_A = 8, \lambda_B = 4$ and $\lambda_A = 4, \lambda_B = 8$ respectively. We find that the relative accuracy is highest when the location with a lower speciation rate is oversampled compared to its representation in the true tree (few of the higher-speciation-rate location-A tips in S5A Fig, and more of the now lower-speciation-rate location-A tips in S5B Fig). In both cases the relative accuracy is highest when the fraction of tips from the lower speciation rate location is 45-65%, whereas due to having a lower speciation rate, that location has fewer tips in the true tree.

Oversampling Recent Migrants

We demonstrate additional results regarding absolute and relative accuracy of downsampled trees with different proportions or recent migrants, where the downsampled trees have 25% and 75% location-A tips instead of 50%. S6 Fig shows the results compared to Fig 2A and Fig 2B, and we observe the same pattern that the accuracy decreases as the proportion of recent migrants increases.

Key Migration Events

We show complementary results about key migration events (KMEs) to Fig 4B and Fig 4AD in S7 Fig, where we count the number of downsampled trees in which a KME of the true tree is obscured, observed or erred for true trees displayed in Fig 1A and Fig 1C respectively.

We perform additional experiments to investigate the absolute accuracy of the parent and the child internal nodes of KMEs.S8 Fig shows the absolute and relative accuracy of the internal nodes of KMEs. We observe that if we sample more location-A tips, then the child nodes of KMEs are more likely to be reconstructed with the wrong location, and the KMEs are likely to be obscured via internal nodes. Similarly, if we sample more location-B tips, then the parent nodes of KMEs are more likely to be reconstructed with

the wrong location, and the KMEs are also likely to be obscured via internal nodes. Therefore, the unbiased geographic sampling performs better in identifying key migration events.

Application to the 2014-2015 Ebola Epidemic

S9 Fig and S10 Fig shows the results of downsampled 80% of the Sierra Leone and Guiena tips respectively. In both cases, thee overall absolute accuracy is high. We obtained 10 downsampled trees for each location and calculate average absolute accuracy. The average absolute accuracy is 99% when downsampling Sierra Leone tips and 94% when downsampling Guinea tips.

Alternative Simulation

We show that the two approaches to simulating geographic sampling, generating true trees then downsampling and downsampling along birth-death processes, give similar results for the absolute accuracy. S11A Fig shows the results of simulating geographic sampling with the first approach. Specifically, we simulate a single true tree with parameters $\lambda = 4$, $\mu = 1$, $\alpha = 0.7$, and we downsample 50 times from the true tree for each proportion of location-A tips. S11B Fig shows the results of the second approach, where we run 50 birth-death processes with sampling using parameters $\lambda = 4$, $\mu = 1$, $\alpha = 0.7$ for each proportion of location-A tips. The function *sim.bdtypes.stt.taxa* in the R package *TreeSim* is used to realize the simulations, and the function produces a downsampled tree for each birth-death process, so we have in total 50 downsampled trees. We observe that the two approaches to simulating geographic sampling produce similar results regarding absolute accuracy.

Epidemic Origin

We also explored how geographic sampling impacts the reconstruction of the location and the time of the root (see S12 Fig). We find that geographic sampling bias can have a dramatic impact on the shows that the reconstructed state of the root depends on the proportion of location-A tips in downsampled trees. Since we begin simulations with a root at location A, the more location-A tips are sampled, the more accurate (i.e. location A) the root location is. S13 Fig also shows that the absolute accuracy of the root location is more accurate when the migration rate of the true tree is low, presumably because more location A tips occur in more monomorphic clades. The reconstructed time of the root also depends on whether there are extinction events or small clades near the root. If there are extinction events (hence tips) or small clades near the root and these are unsampled, then the reconstructed time of the root can be inaccurate.
