# Supplementary figures and images for: The impact of sampling bias on viral phylogeographic reconstruction
Source: PLOS Glob Public Health. 2022 Sep 28;2(9):e0000577. doi: 10.1371/journal.pgph.0000577 (PMC10021582; doi:10.1371/journal.pgph.0000577)

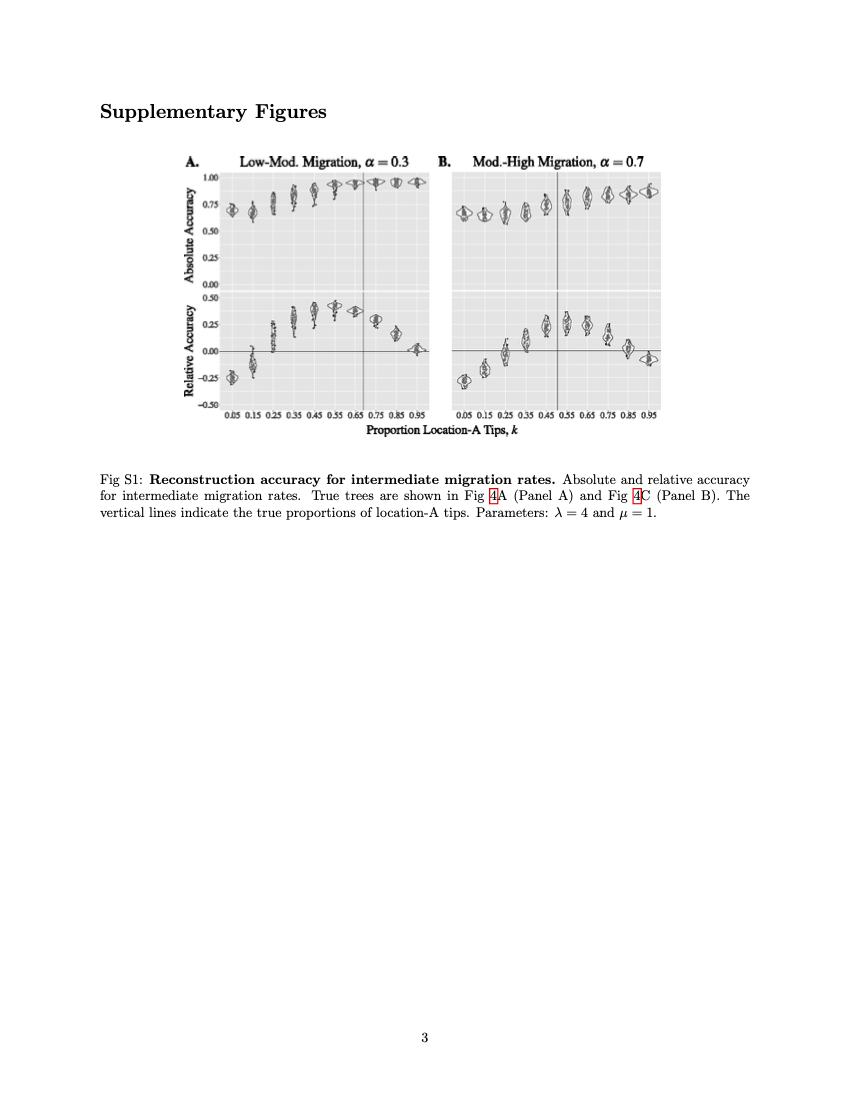

Supplement: S1 Fig — (TIFF) [file pgph.0000577.s002.tiff]

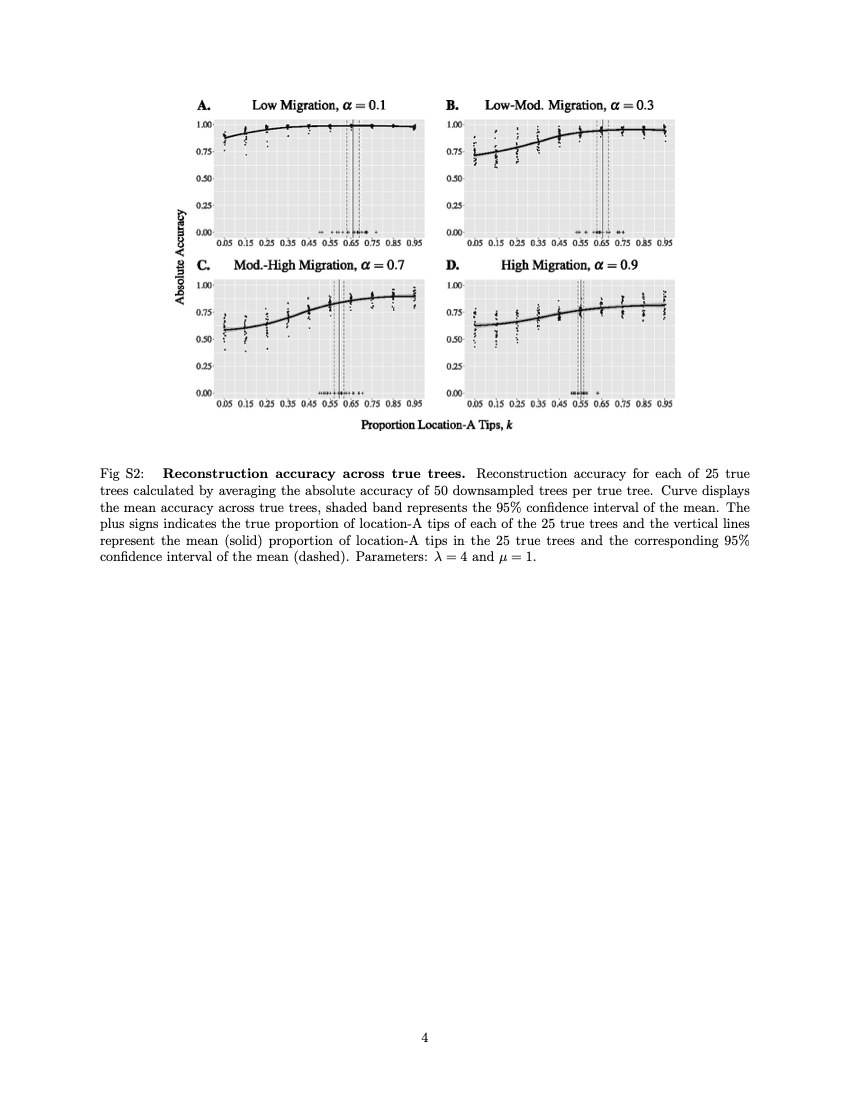

Supplement: S2 Fig — (TIFF) [file pgph.0000577.s003.tiff]

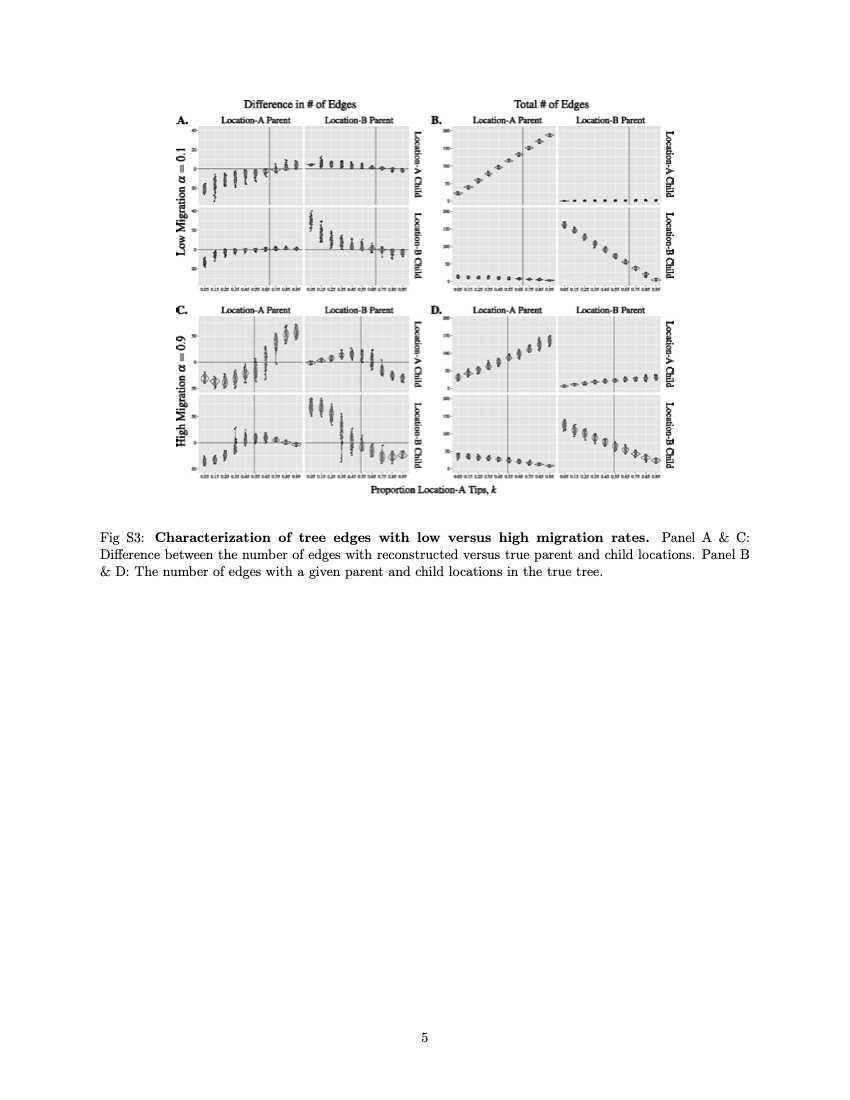

Supplement: S3 Fig — (TIFF) [file pgph.0000577.s004.tiff]

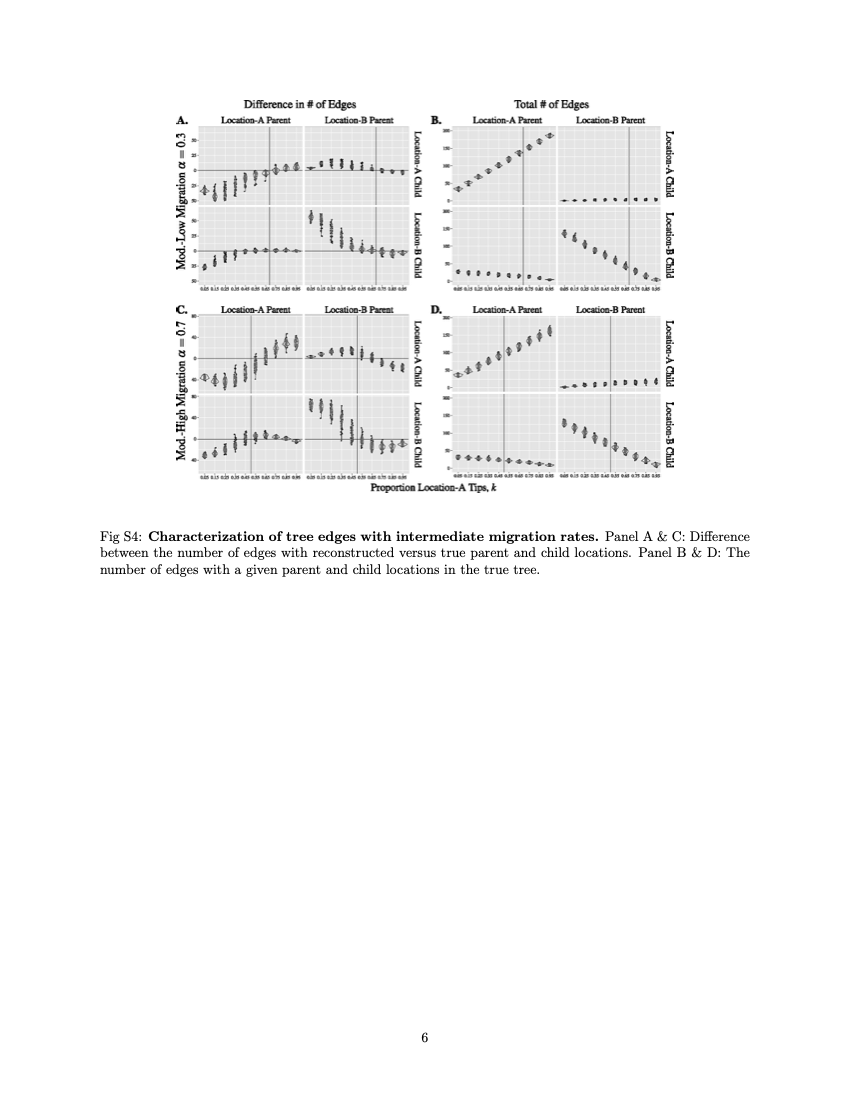

Supplement: S4 Fig — (TIFF) [file pgph.0000577.s005.tiff]

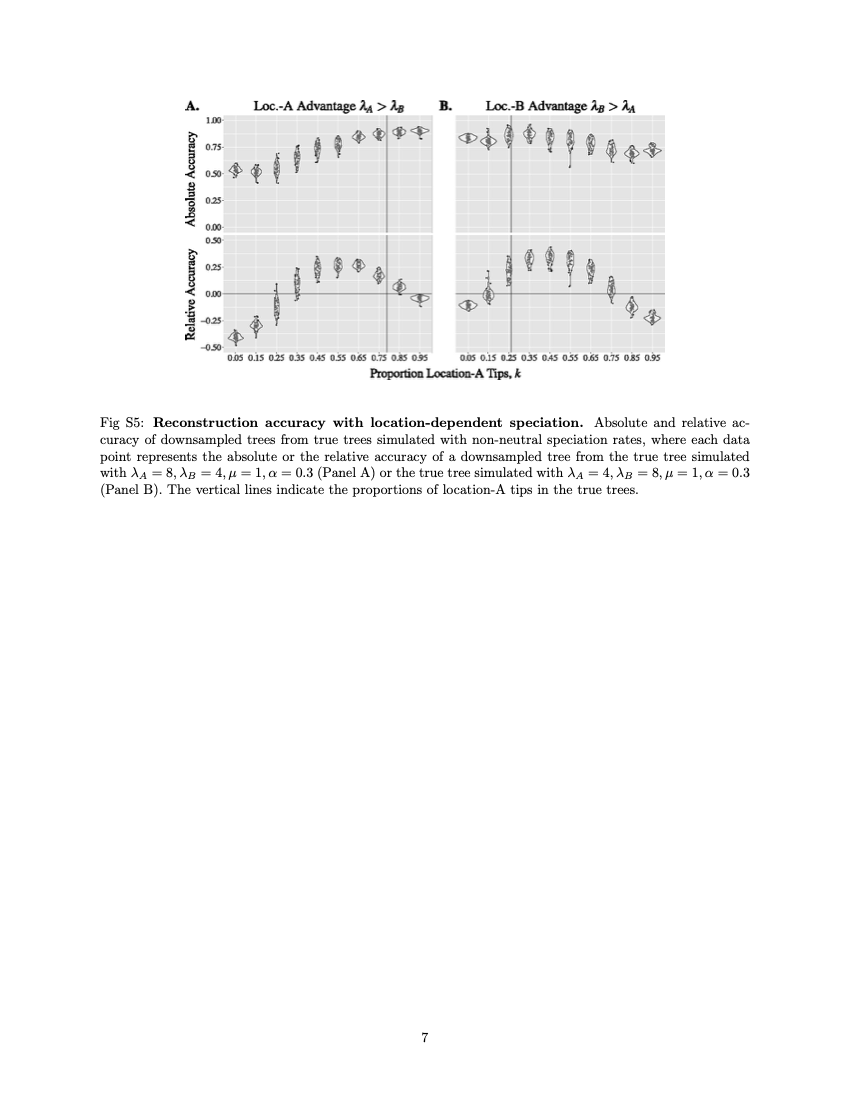

Supplement: S5 Fig — (TIFF) [file pgph.0000577.s006.tiff]

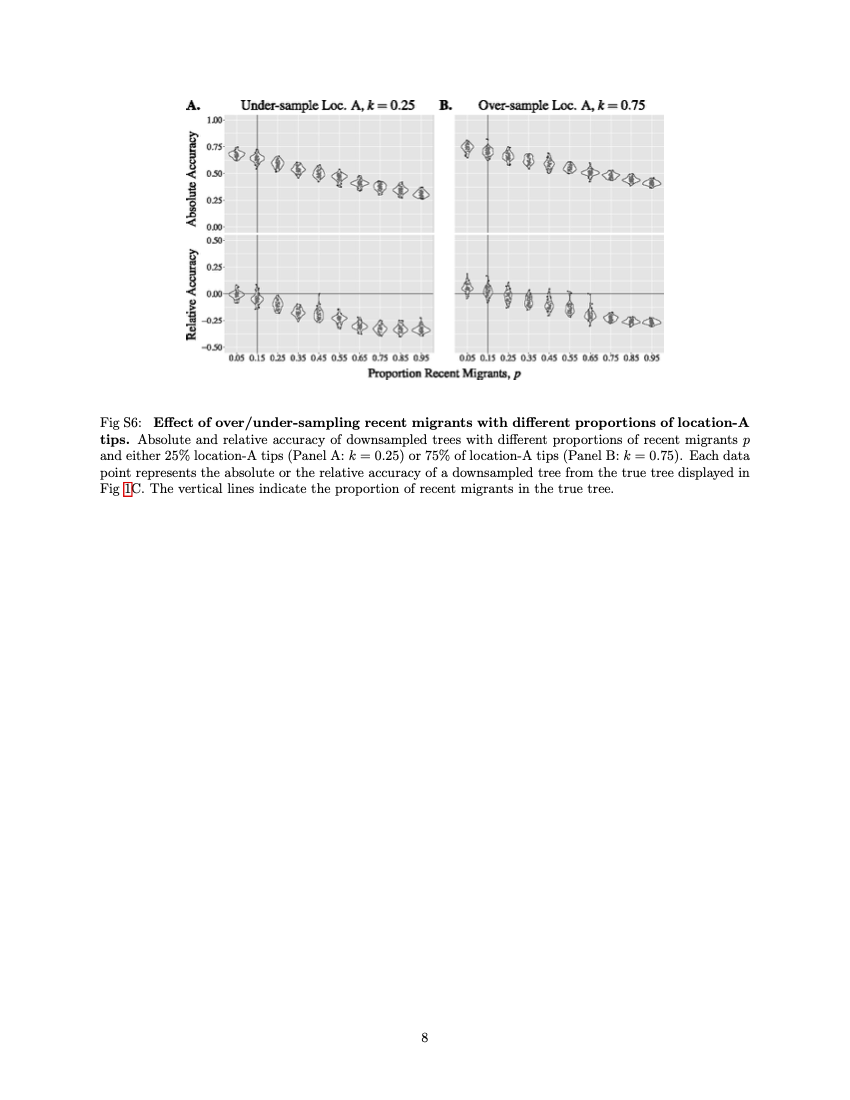

Supplement: S6 Fig — (TIFF) [file pgph.0000577.s007.tiff]

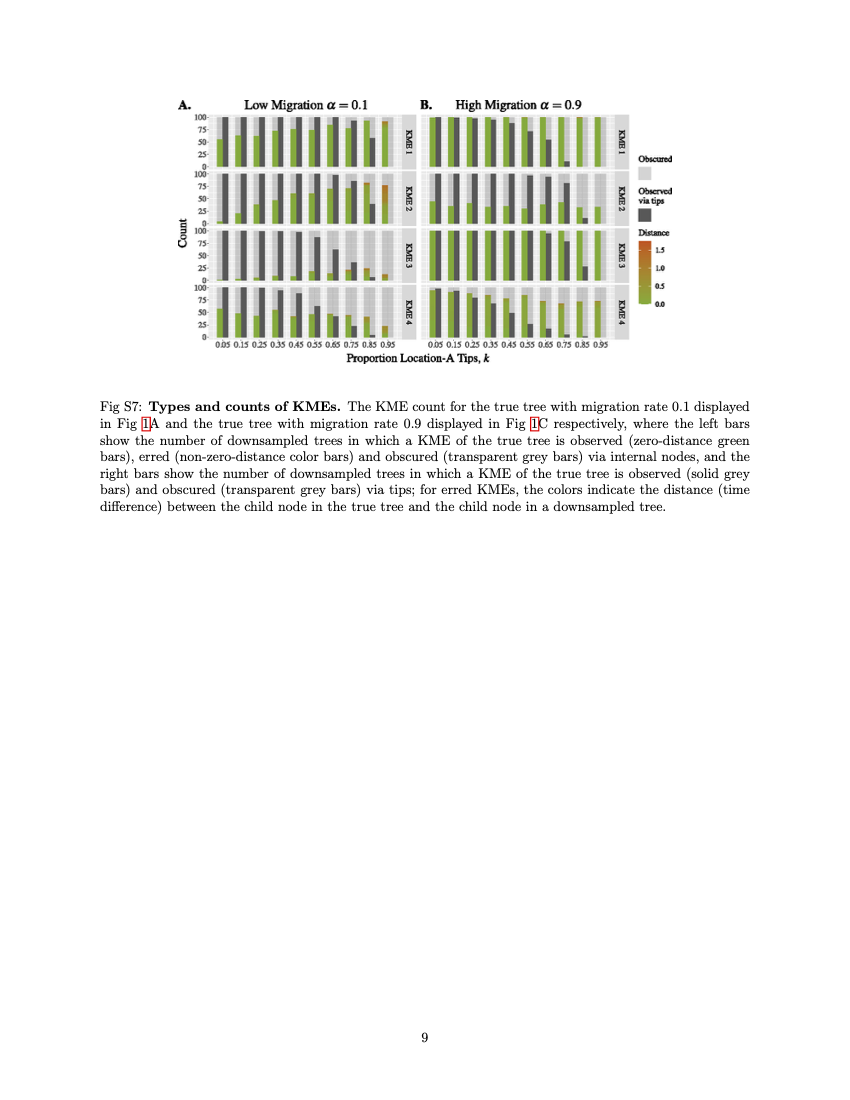

Supplement: S7 Fig — (TIFF) [file pgph.0000577.s008.tiff]

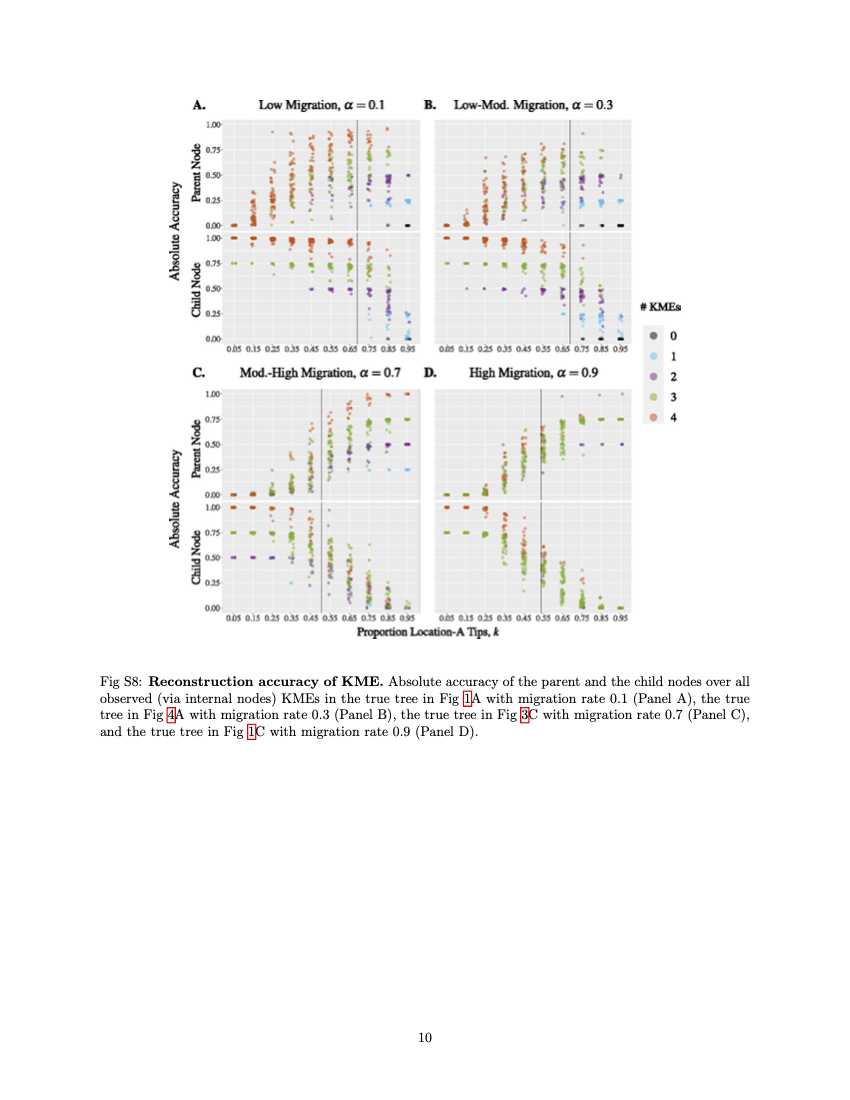

Supplement: S8 Fig — (TIFF) [file pgph.0000577.s009.tiff]

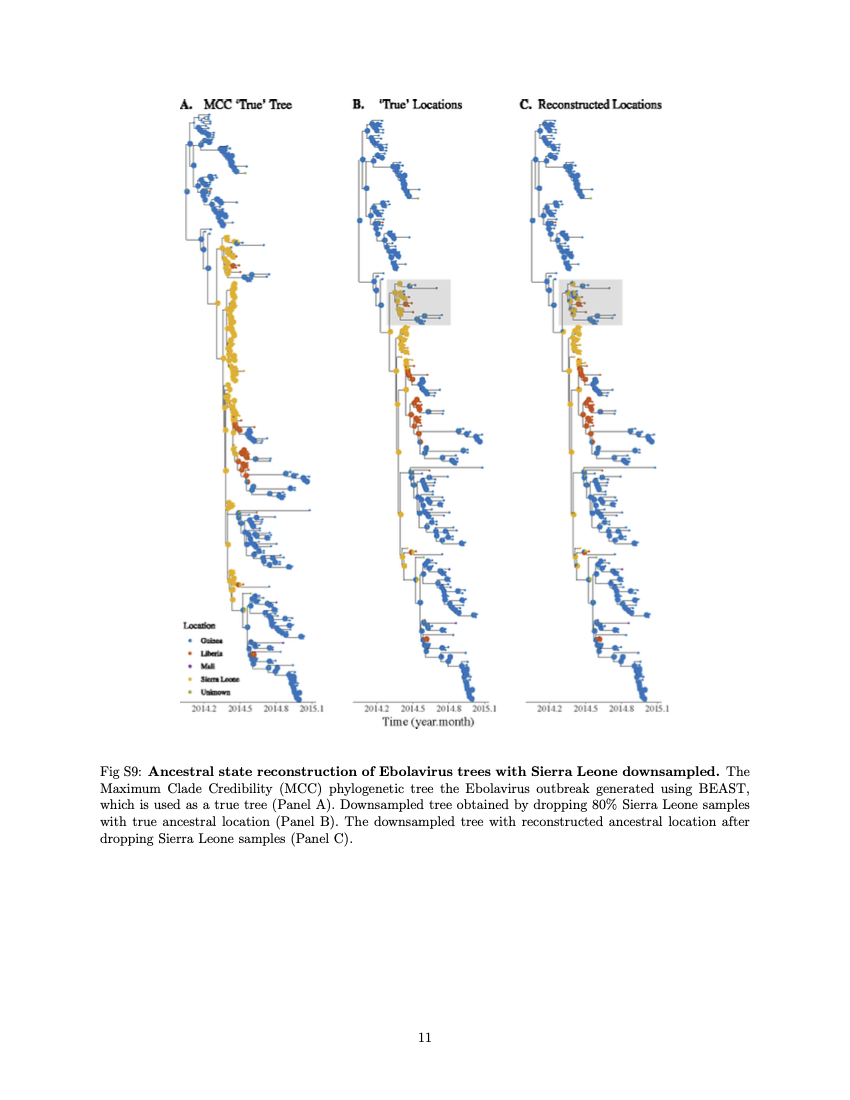

Supplement: S9 Fig — (TIFF) [file pgph.0000577.s010.tiff]

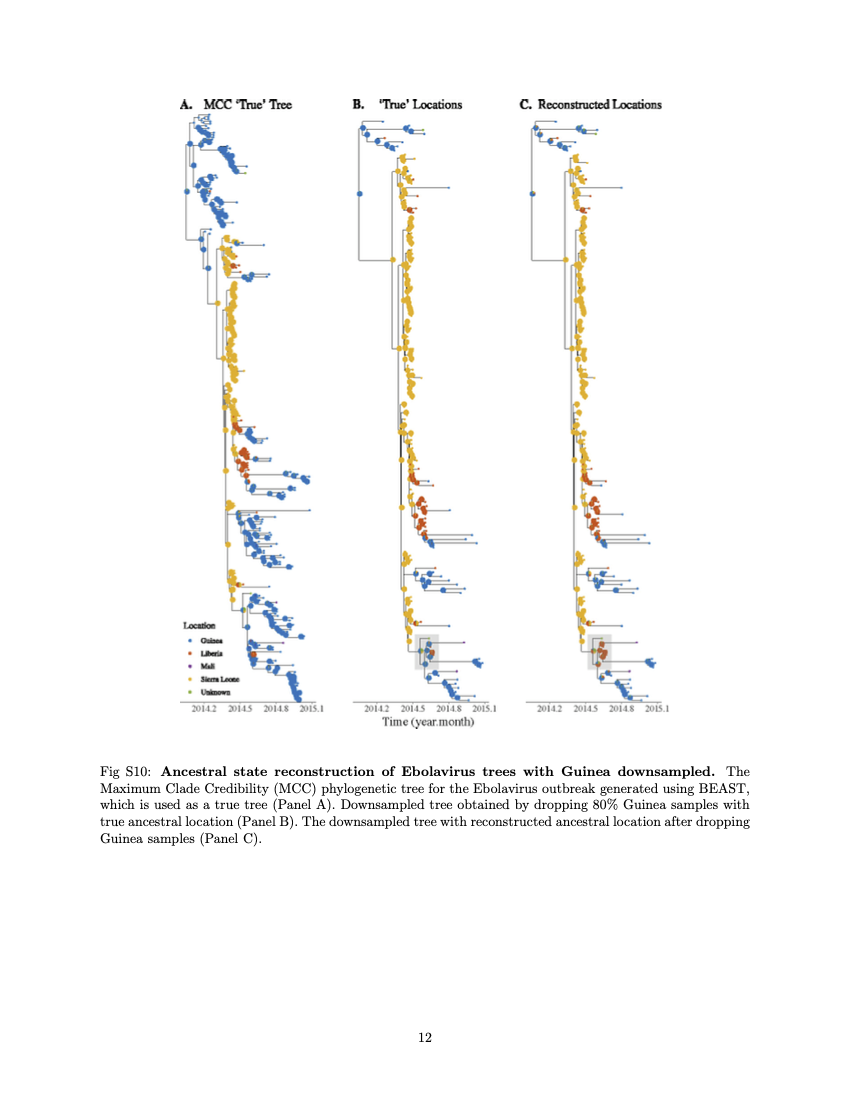

Supplement: S10 Fig — (TIFF) [file pgph.0000577.s011.tiff]

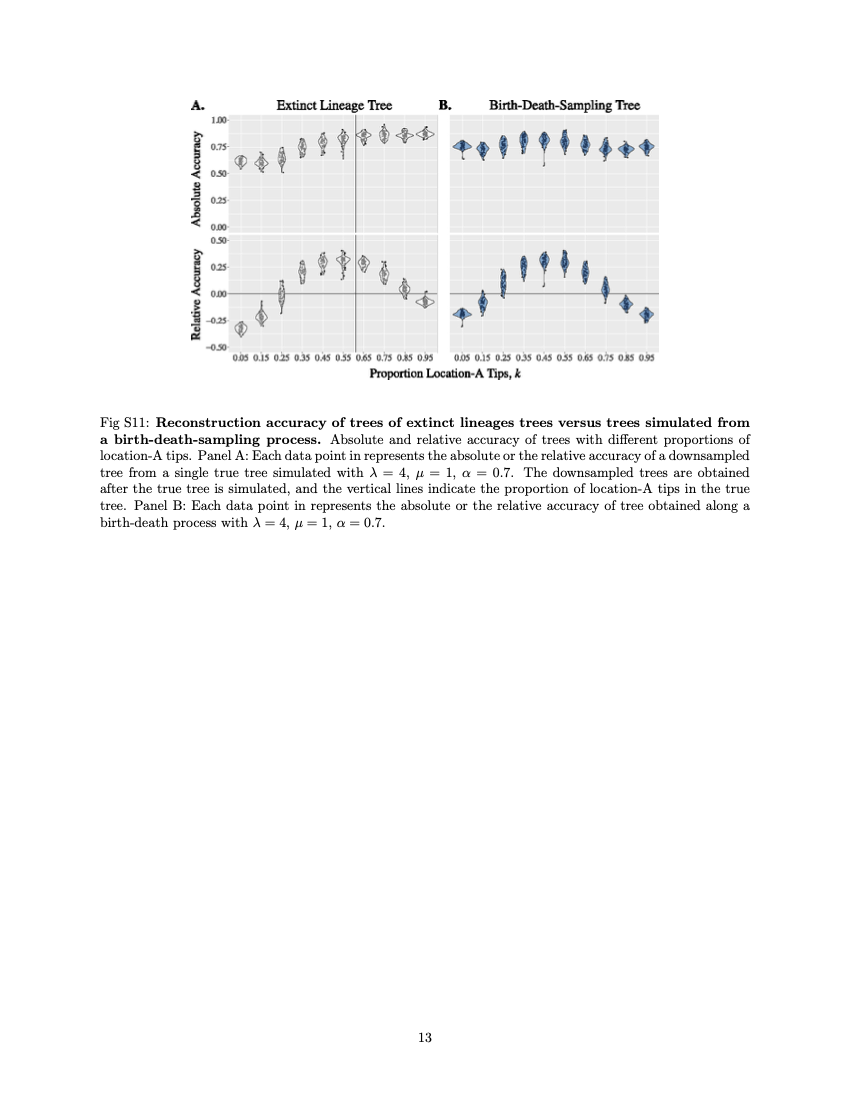

Supplement: S11 Fig — (TIFF) [file pgph.0000577.s012.tiff]

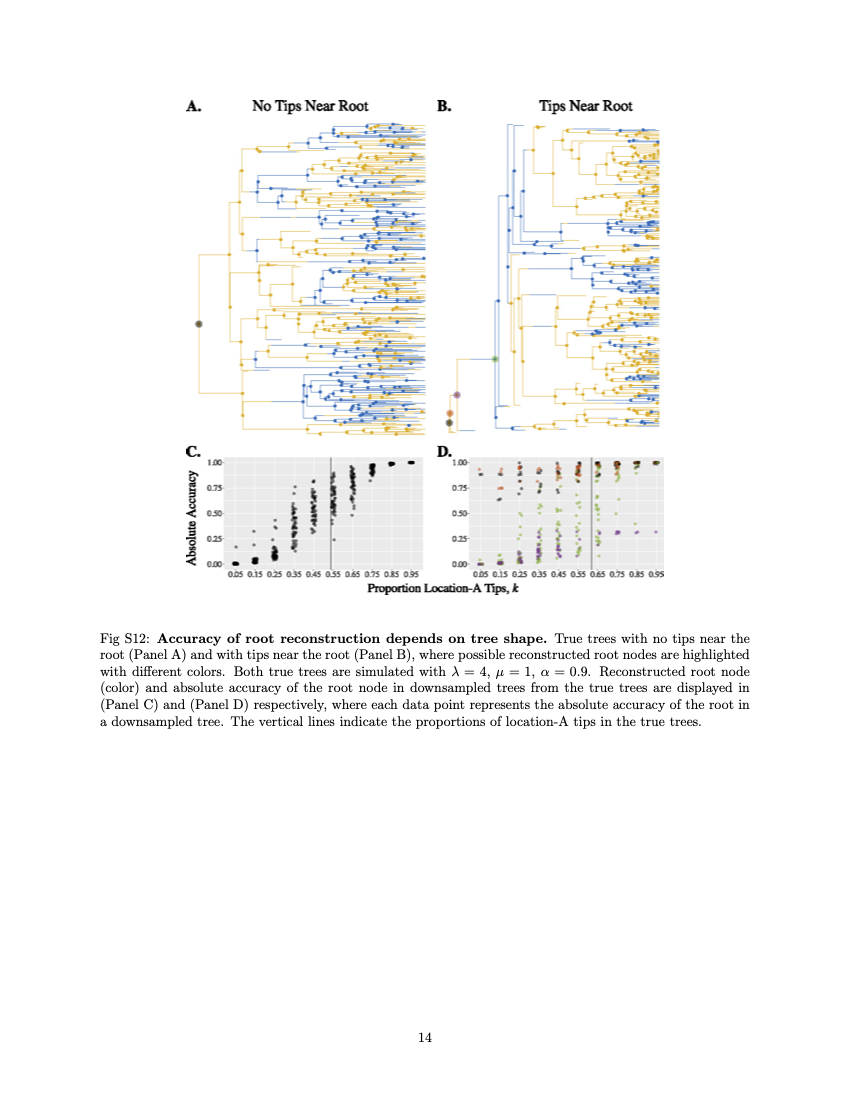

Supplement: S12 Fig — (TIFF) [file pgph.0000577.s013.tiff]

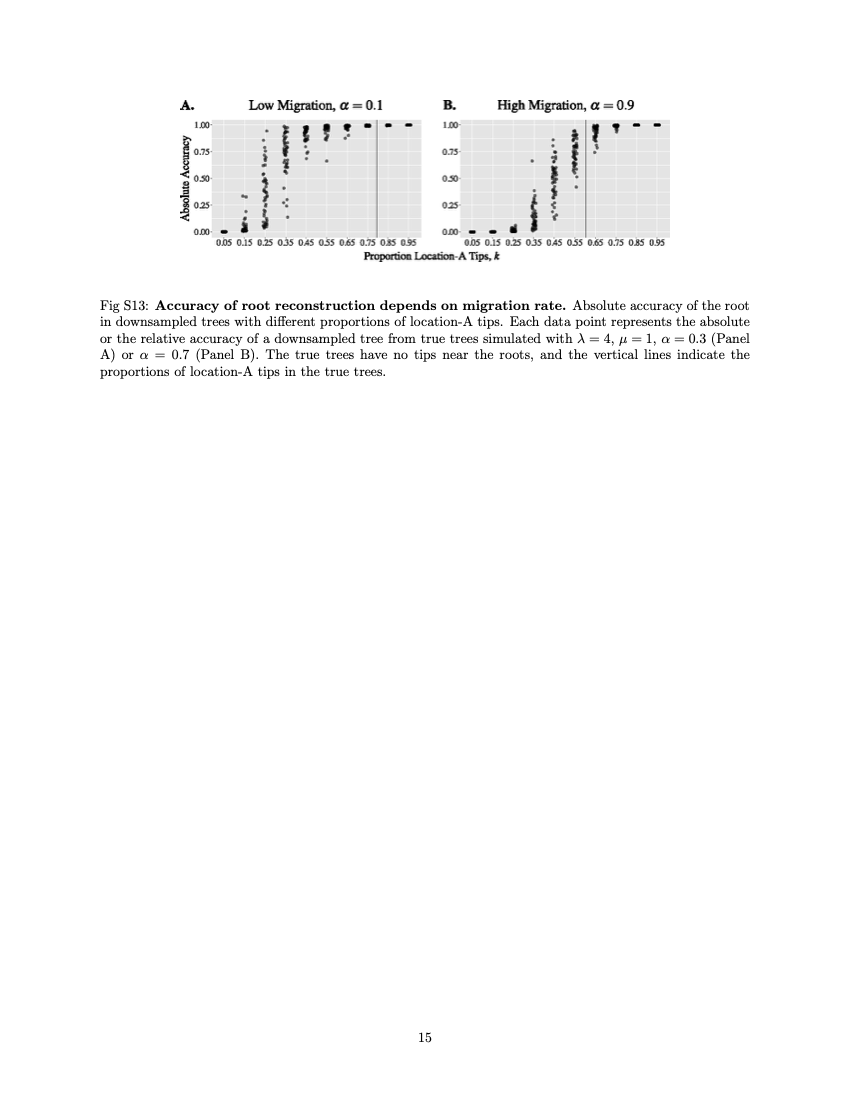

Supplement: S13 Fig — (TIFF) [file pgph.0000577.s014.tiff]
