# Supplementary material for: The impact of sampling bias on viral phylogeographic reconstruction
Source: PLOS Glob Public Health. 2022 Sep 28;2(9):e0000577. doi: 10.1371/journal.pgph.0000577 (PMC10021582; doi:10.1371/journal.pgph.0000577)
Supplement: Attachment — Submitted filename: Response.pdf [file pgph.0000577.s015.pdf]

# Reviewer 1:

- Despite mentioning the source of the data in the data availability statement, I would suggest mentioning the source of the sequence data which subsequent analyses are based on at the beginning of the M & M section, otherwise the reader is unsure about whether the sequences have been produced in this study or retrieved from an online repository.
  - Line 114: We have added a reference to the code.
- line 218: '...the there..', please resolve
  - Line 219: We have revised this sentence.
- line 305: delete space between there and of
  - Line 306: We have made this change.
- line 337 and onwards: there's something wrong with this sentence, please correcct/rephrase
  - Line 338: Thank you for catching this. We have reworded these few sentences
- Fig4 legend: low-moderate what? I think there is something missing here. It's 'whether' here not 'weather', right?
  - We have corrected both of these
- line 425: summarises
  - Line 428: We have change this.
- line 486: delete one 'are'
  - Line 497: We have done this.
- line 505: delte one 'to be'
  - We have done this.
- page 26: 'Here we simulate under with...' appears grammatically odd to me.
  - We have reworded this to be "Here, we simulate trees with"
- Fig S5: 'Reconstruction accuracy for with...' appears grammatically odd to me.
  - We have deleted the 'for'

# Reviewer 2:

- I just suggest that the color palette in figures 5, S9 and S10 be changed, allowing for a better distinction between the locations.
  - Thank you for the suggestion. We have done this.
